# Exploring the pre-immune landscape of antigen-specific T cells

**DOI:** 10.1186/s13073-018-0577-7

**Published:** 2018-08-25

**Authors:** Mikhail V. Pogorelyy, Alla D. Fedorova, James E. McLaren, Kristin Ladell, Dmitri V. Bagaev, Alexey V. Eliseev, Artem I. Mikelov, Anna E. Koneva, Ivan V. Zvyagin, David A. Price, Dmitry M. Chudakov, Mikhail Shugay

**Affiliations:** 1 Department of Genomics of Adaptive Immunity, IBCH RAS, Moscow, Russia; 2 Division of Infection and Immunity, Cardiff University School of Medicine, Cardiff, UK; 3 Department of Molecular Technologies, Pirogov Russian National Research Medical University, Moscow, Russia; 4 Center for Data-Intensive Biomedicine and Biotechnology, Skoltech, Moscow, Russia; 5 Central European Institute of Technology, CEITEC, Brno, Czech Republic; 6 Systems Immunity Research Institute, Cardiff University School of Medicine, Cardiff, UK

**Keywords:** Antigen, Immune repertoire, Immunogenicity, T cell receptor

## Abstract

**Background:**

Adaptive immune responses to newly encountered pathogens depend on the mobilization of antigen-specific clonotypes from a vastly diverse pool of naive T cells. Using recent advances in immune repertoire sequencing technologies, models of the immune receptor rearrangement process, and a database of annotated T cell receptor (TCR) sequences with known specificities, we explored the baseline frequencies of T cells specific for defined human leukocyte antigen (HLA) class I-restricted epitopes in healthy individuals.

**Methods:**

We used a database of TCR sequences with known antigen specificities and a probabilistic TCR rearrangement model to estimate the baseline frequencies of TCRs specific to distinct antigens epitopespecificT-cells. We verified our estimates using a publicly available collection of TCR repertoires from healthy individuals. We also interrogated a database of immunogenic and non-immunogenic peptides is used to link baseline T-cell frequencies with epitope immunogenicity.

**Results:**

Our findings revealed a high degree of variability in the prevalence of T cells specific for different antigens that could be explained by the physicochemical properties of the corresponding HLA class I-bound peptides. The occurrence of certain rearrangements was influenced by ancestry and HLA class I restriction, and umbilical cord blood samples contained higher frequencies of common pathogen-specific TCRs. We also identified a quantitative link between specific T cell frequencies and the immunogenicity of cognate epitopes presented by defined HLA class I molecules.

**Conclusions:**

Our results suggest that the population frequencies of specific T cells are strikingly non-uniform across epitopes that are known to elicit immune responses. This inference leads to a new definition of epitope immunogenicity based on specific TCR frequencies, which can be estimated with a high degree of accuracy in silico, thereby providing a novel framework to integrate computational and experimental genomics with basic and translational research efforts in the field of T cell immunology.

**Electronic supplementary material:**

The online version of this article (10.1186/s13073-018-0577-7) contains supplementary material, which is available to authorized users.

## Background

The availability of huge volumes of repertoire sequencing (RepSeq) [1] data and a growing curated list of T cell receptor (TCR) sequences with known antigen specificities [2] have enabled quantitative exploration of the adaptive immune system. Previous large-scale studies of immune repertoire structure in health and disease have been limited in the main to analyses of basic parameters, such as repertoire diversity and somatic rearrangement patterns incorporating variable (V), diversity (D), and joining (J) segments of the TCR [3–6]. However, it is now possible to extract potentially more useful information from these rich datasets by stratifying for antigen specificity, as exemplified recently in the settings of cytomegalovirus (CMV) infection [7] and ankylosing spondylitis [8].

Theoretical [9] and experimental [10] studies have indicated that the ability of the T cell repertoire to recognize any novel antigen is essentially determined by the frequency of antigen-specific clonotypes prior to immune challenge. The emergence of sensitive major histocompatibility complex (MHC) multimer staining protocols has further permitted the accurate measurement of specific T cell populations in the naive pool [11]. Using this approach, it has been shown that the absolute numbers of specific T cells in the pre-immune repertoire vary greatly across different epitopes, yet remain largely conserved across individuals [12, 13]. Moreover, the frequency of antigen-specific T cells in the naive pool determines both the magnitude and the kinetics of the cognate immune response [10].

Recent estimates suggest that naïve T cell clone can be as small as ~ 5 cells, which constitutes a negligible fraction of ~ 3 × 10^11^ T cells in the human body [14]. This leads to the observation that naive T cells specific for certain antigens are often present at very low frequencies, in some cases around one cell per million sampled T cells [10], which makes them hard to detect reliably, even using modern high-throughput RepSeq techniques. Accurate quantification via flow cytometry is a similarly challenging task [15]. However, recently developed computational methods based on probabilistic models of the VDJ rearrangement process have allowed surprisingly precise estimates of generation frequency for individual nucleotide [16] and amino acid [17] sequences, which in turn dictate the antigen specificity of a TCR repertoire. These approaches can be used in conjunction with RepSeq data and TCR specificity annotation to characterize the pre-immune landscape of antigen-specific T cells.

We hypothesized that a growing knowledge base of antigen-specific TCR sequences, together with recent advances in RepSeq techniques and theoretical models of the TCR repertoire formation might allow in silico enumeration of T cells specific for different epitopes. An analytical framework that integrates these various datasets could provide quantitative answers to several intriguing questions regarding the organization of the adaptive immune system. For example, one could ask if major differences exist among epitope-specific T cell frequencies and how any such differences relate to the biochemical and structural properties of the targeted epitopes. One could also ask if specific T cell frequencies vary depending on the origin of T cells (for example, between cells derived from peripheral and umbilical cord blood) and individual ancestry. In addition, one could define the concept of immunogenicity in terms of the ability of the adaptive immune system to field specific T cells as a function of individual and population-level biases in repertoire structure determined by stochasticity and variability in the VDJ rearrangement process.

In this study, we report the first comprehensive analysis of baseline frequencies and population incidence rates for TCRs with known specificities that target human leukocyte antigen (HLA) class I-restricted epitopes derived from eight different pathogens. We developed a computational model that accurately predicts the baseline frequency of individual antigen-specific TCR amino acid variants curated in a publicly available database (VDJdb). This model was verified using 859 unfiltered RepSeq datasets from healthy donors [3, 7]. Accordingly, our computational framework provided a solid basis to quantify the population frequencies of antigen-specific TCRs, explore the phenomenon of shared (“public”) clonotypes [18–20], and perform in silico analyses of various factors that shape the pre-immune repertoire. Using this approach, we further assessed the impact of ancestry and HLA class I type on antigen-specific T cell frequencies and characterized the specificity landscape in umbilical cord blood samples, which allowed unique insights into a convergent and highly stable “core” repertoire of naive T cells [3]. Finally, we mined a large dataset of epitopes with known immunogenicity scores [21] to derive a probabilistic measure of antigenic potential. This novel variable was used to refine our understanding of epitope specificity and develop a hierarchical view of adaptive immune responses.

## Methods

### Datasets and pre-processing

We used two publicly available human TCRβ RepSeq datasets: (i) data from Emerson et al. [7] (available at [https://clients.adaptivebiotech.com/immuneaccess]) and (ii) data from Britanova et al. [3] (available at [https://zenodo.org/record/826447]). TCR reads from Emerson et al. were re-mapped using MiXCR software v2.1.5 with default settings (no clonotype assembly was performed, only read mapping using the MiXCR Align routine) [22] to provide V, D, and J segment assignments consistent with Britanova et al. and IMGT nomenclature [23]. The resulting clonotype tables were then processed using VDJtools software v1.1.6 with default settings [6]. Samples were corrected for sequencing errors (VDJtools Correct routine), split into coding and non-coding clonotypes (VDJtools FilterNonFunctional routine), and VJ segment use was computed to model TCR rearrangements (VDJtools CalcSegmentUsage routine). Clonotypes were pooled by CDR3 amino acid sequence using the VDJtools PoolSamples routine to determine population frequency and the total number of TCR nucleotide sequence variants. Identical variants were counted if they were observed in different individuals. The number of nucleotide variants was used as a measure of baseline TCR frequency. Although expanded memory T cells occupy a major fraction of the repertoire, they account for only a minor fraction of unique variants [3]. Analyses based on counting unique rearrangements are therefore relatively unbiased by clone size.

### T cell repertoire annotation

Human TCRβ sequences known to bind certain HLA class I-restricted epitopes were obtained from the VDJdb database [2]. CDR3 amino acid sequence matching that allowed at most one amino acid substitution and no indels was used to assign antigen specificities to RepSeq data. Of note, exact matching that required CDR3 sequence and V/J gene identity resulted in far fewer hits, rendering this approach unfeasible for currently available TCR datasets. Application of this procedure to the VDJdb data increased matches with concordant antigen by an additional ~ 20%, while allowing more substitutions linearly increased the number of discordant matches, which became greater than the concordant match frequency at three substitutions [2]. This procedure is stricter than those proposed in other analyses [24, 25]. The method used by Dash et al. allowed both substitutions and indels, while the method used by Glanville et al. operated with k-mers, allowing several substitutions.

**Fig. 1:**
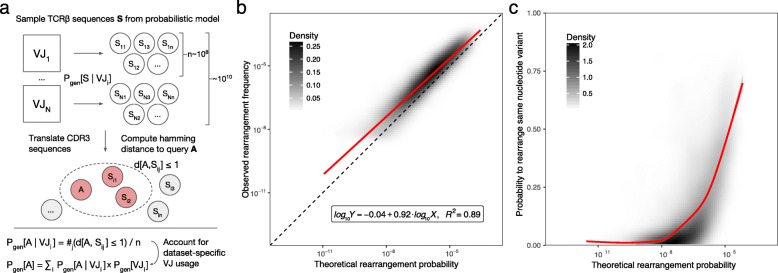
Estimating baseline T cell frequencies using a VDJ rearrangement model. **a** Schematic description of the TCRβ baseline frequency estimator. CDR3 sequences were sampled from the pre-trained probabilistic model of Murugan et al. for each VJ segment combination, translated, and matched to a given CDR3 sequence (allowing at most one amino acid substitution, see the “Methods” section) to estimate its theoretical rearrangement probability. Resulting probabilities were corrected for the sample-specific VJ segment frequency profile. **b** The observed (*Y*-axis) versus estimated (*X*-axis) rearrangement frequencies for 6853 human TCR sequences with known antigen specificities selected from VDJdb in 786 immune repertoire samples from Emerson et al. containing 151,020,646 unique rearrangements (identical TCRβ nucleotide sequences observed in different donors were counted as distinct). Observed frequencies were computed as the total number of unique rearrangements encoding a given CDR3 amino acid sequence in the pooled dataset (with at most one substitution) divided by the total number of unique rearrangements. The red line displays the linear model fit for log-transformed frequencies. **c** Density plot showing the probability of rearranging the same nucleotide sequence in different individuals versus the theoretical rearrangement probability for VDJdb TCR variants (amino acid sequences). The red curve displays the smoothing fit

It is important to note that our method does not require V or J segment matches, yet in most cases, it also does not allow mismatches in germline-encoded regions of the CDR3. The latter can determine the J segment accurately and narrow the list of possible V segment variants. The implicit matching of V and J segments resulting from our annotation method is reflected in the strong correlation observed between annotated TCR sequencing data and the TCR rearrangement model (described below) that uses both V and J segment information (see Fig. 1b).

### Estimating rearrangement probabilities for TCR amino acid variants

The probabilistic model for TCR sequence generation was described previously [26]. Briefly, the probability of recombination scenario is represented as the product of probabilities of distinct events in the VDJ recombination process:

1 $$ {\displaystyle \begin{array}{c}{P}_{\mathrm{rearr}}^{\beta }(r)=P(V)P\left(D,J\right)P\left(\left.\mathrm{del}V\right|V\right)P\left(\mathrm{ins} VD\right)\\ {}\times P\left(\mathrm{del} Dl,\left.\mathrm{del} Dr\right|D\right)P\left(\mathrm{ins} DJ\right)P\left(\left.\mathrm{del}J\right|J\right).\end{array}} $$

2 $$ {P}_{gen}(n)=\sum \limits_{r\in {r}_n}{P}_{\mathrm{rearr}}^{\beta }(r) $$

3 $$ {P}_{gen}(a)=\sum \limits_{n\in {n}_a}{P}_{gen}(n) $$

where P(V) and P(D,J) are the probabilities of V and D,J pair choices, P(delV|V) is the probability of a certain number of 3′ nucleotide deletions from the V segment at the VD junction, P(delJ|J) is the probability of a certain number of 5′ nucleotide deletions from the J segment at the DJ junction, P(delDl,delDr) accounts for 3′ and 5′ D segment deletions, and P(insVD) and P(insDJ) are the probabilities of certain insertion sequences at the VD and DJ junctions, respectively. Probability tables used in this study were identical to those provided in [26], with the exception of P(V) and P(D,J), which were obtained by computing the V/J frequencies of non-functional clonotypes in the Emerson et al. and Britanova et al. datasets. The latter were calculated to account for potential V/J biases arising from methodological differences in the procedure used to generate TCR amplicon libraries (Emerson et al. used multiplexed polymerase chain reactions, and Britanova et al. used 5′ rapid amplification of cDNA ends).

To calculate the generative probability for a given nucleotide sequence, we summed the probabilities of all possible scenarios that can generate that nucleotide sequence (see Eq. 2). Amino acid sequence generation probability was then computed as the sum of probabilities of all possible underlying nucleotide sequences (see Eq. 3). Exact calculation of the probability of generating a particular amino acid sequence is computationally expensive, mostly due to the presence of a short D segment, so we used the previously described Monte Carlo approach [17]. Briefly, we generated a large set of possible rearrangements from the model, translated the resulting nucleotide sequences, and counted the number of matches to the CDR3 amino acid sequence of interest, allowing a fixed number of mismatches.

It has been shown previously that the profiles of randomly added and deleted nucleotides are very stable across repertoires sequenced using different technologies, in contrast to identification of the V and J segments [16], which are subject to amplification bias during library preparation. This leads to differences in the P(V) and P(D,J) distributions, which can be accounted for by computing P(CDR3aa) in two steps: (i) compute P(CDR3aa|V,J) by simulating recombination scenarios for a fixed VJ combination (J unambiguously determines possible D); and (ii) calculate P(CDR3aa) as a sum of P(CDR3aa|V,J) times P(V,J), where P(V,J) is estimated from non-functional sequences in the dataset of interest. In this study, we simulated 10^8^ recombination scenarios for each VJ combination, generating more than 10^10^sequences in total. We then scaled the estimated frequencies by VJ usage in the corresponding RepSeq dataset. Of note, the final probabilities can fall below 10^−10^, because some VJ combinations have a frequency of less than 10^−2^.

### Analysis of amino acid features

The physicochemical properties of CDR3 loops and peptide epitopes were estimated using sums of ten Kidera factors (see [27] for more details and corresponding values) across all residues. Kidera factors were originally derived as principal components of various physicochemical properties of individual amino acids and encode features such as volume and hydropathy (as determined by the origin of the largest term in a factor). In our analysis, we computed the Pearson correlation between raw factor values and the variable of interest (e.g., rearrangement probability) and used an ANOVA test for the values of a given Kidera factor partitioned into four quantiles. The partitioning was done based on the whole spectrum of Kidera factor sums observed for all VDJdb epitopes with the first (Q1) and last (Q4) quantiles corresponding to the highest and lowest factor values, respectively.

### Statistical analysis

All statistical testing was performed in R using standard packages for *T* test, ANOVA, Mann–Whitney *U* test, and Kolmogorov–Smirnov test. R markdown templates for all analysis steps are available at [https://github.com/antigenomics/public-epitope].

## Results

### Modelling baseline frequencies of specific TCR amino acid sequences

It has been shown previously that the chance of a certain TCR nucleotide sequence being produced by the VDJ rearrangement process can be efficiently recaptured with a probabilistic model that considers V, D, and J gene choices, the number of bases trimmed from the rearranged germline sequences, and the number and composition of random insertions [26]. This model can be applied reliably to a given TCR repertoire using an expectation maximization algorithm, and the results are extremely stable across individuals [16, 26]. However, estimating the probability of TCR variants and their amino acid translations requires traversing a large tree of possible rearrangement scenarios, which can be computationally inefficient. We therefore chose to compute approximate probabilities using the Monte Carlo method, which operates in a two-step manner: (i) it counts the expected number of matches to a given CDR3 amino acid sequence within a given V(D)J combination by sampling rearrangements using corresponding V/D/J trimming and random insert probabilities [26] and (ii) it scales match frequencies to account for a specific V(D)J combination frequency profile in a given dataset and computes the final probability value by summarizing frequencies across different V(D)J combinations (see the “Methods” section and Fig. 1a). This method was used to estimate the probability of observing a certain TCR beta chain (TCRβ) CDR3 amino acid sequence with a maximum discrepancy of one amino acid substitution, which in turn was used as a proxy to estimate specific T cell frequency throughout this study.

Baseline frequencies of TCR variants estimated using this method were in good agreement with those observed in a dataset of 786 repertoires (Fig. 1b). The intercept of the model was close to zero (− 0.04 ± 0.03) after correcting for the percentage of non-coding sequences (either out-of-frame or containing a stop codon) generated by the probabilistic model (24.3 ± 0.1%). A slope of 0.920 ± 0.005 could be attributed to sampling effects, because the frequencies observed in the real dataset exhibited a lower bound of 10^−7^ to 10^−8^, which was much higher than the corresponding range in the theoretical model.

The case where multiple TCR nucleotide sequences encode the same TCR amino acid sequence (also known as convergent recombination) has previously been linked to the phenomenon of “public” TCRs, which are shared across multiple individuals [18]. As can be seen from Fig. 1c, this process was also observed for TCR variants with high rearrangement frequencies, in some instances exceeding previous estimates. Moreover, for the most frequent TCR amino acid variants, as many as three in four separate rearrangement events generated the same TCR nucleotide sequence.

### Rearrangement probabilities and population frequencies vary greatly across T cells specific for different antigens

**Fig. 2:**
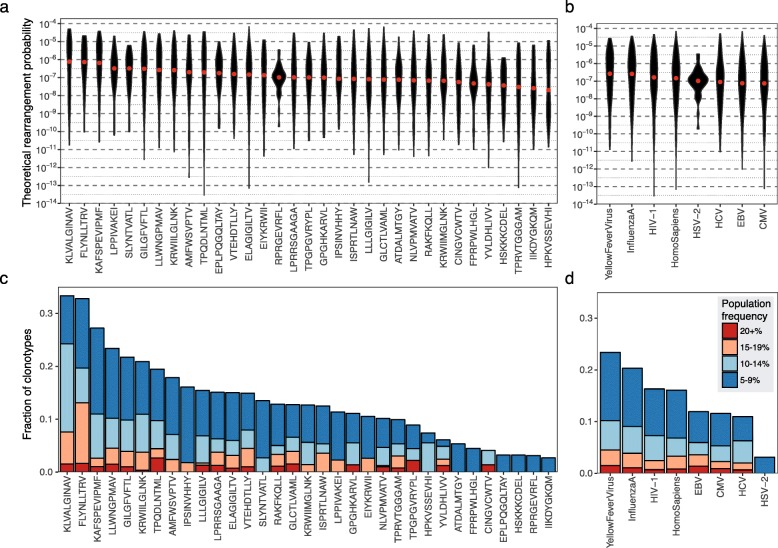
Rearrangement probabilities and population frequencies of TCRs specific for different antigens. **a** Estimated rearrangement probabilities for TCRs specific for 33 different HLA class I-restricted epitopes. Only epitopes associated with at least 30 different TCR amino acid sequences were selected from VDJdb (*n* = 5623 TCRs). The distribution of theoretical rearrangement probabilities is shown using violin plots; red dots indicate the median rearrangement probabilities. The variance of specific TCR frequencies across different epitopes is highly significant (*P* < 10^−27^, ANOVA for log probabilities). **b** As in **a**, but the TCR sequences are grouped by epitope origin. The difference in rearrangement probabilities among epitopes grouped by origin is also highly significant (*P* < 10^−11^, ANOVA for log probabilities). **c** Fractions of clonotypes specific for different epitopes showing population frequencies of 5–9%, 10–14%, 15–19%, or 20%+ in 786 immune repertoire samples from Emerson et al. **d** As in **c**, but grouped by epitope origin

Next, we applied this model to explore frequency differences across distinct antigen-specific T cell populations. As can be seen from Fig. 2a, the median frequencies of TCR variants associated with different epitopes varied significantly, and the difference between the highest and lowest associated frequencies was almost two orders of magnitude. Nonetheless, the intra-epitope frequency variance reached six orders of magnitude, suggesting that each epitope featured both public and rare antigen-specific TCRs. These differences were also present when TCR variants were grouped by epitope origin (Fig. 2b). Interestingly, TCR variants specific for CMV or Epstein–Barr virus (EBV) were the least frequent, ruling out the hypothesis that the VDJ rearrangement machinery is biased towards targeting common pathogens [28].

Of note, these results translated into population frequencies of specific TCR variants. The fraction of individuals with a specific TCR variant shown in Fig. 2c, d closely mirrored the theoretical frequencies shown in Fig. 2a, b, and the Spearman rank correlation coefficient between median rearrangement probability and the fraction of epitope-specific TCRs found in at least 5% samples was *ρ* = 0.71 (*P* = 4 × 10^−6^). This finding suggests that differences in baseline frequencies resulting from intrinsic features of the VDJ recombination machinery may have a profound effect on immunity at the population level.

### Epitope sequence features can predict the population frequency of specific T cells

**Fig. 3:**
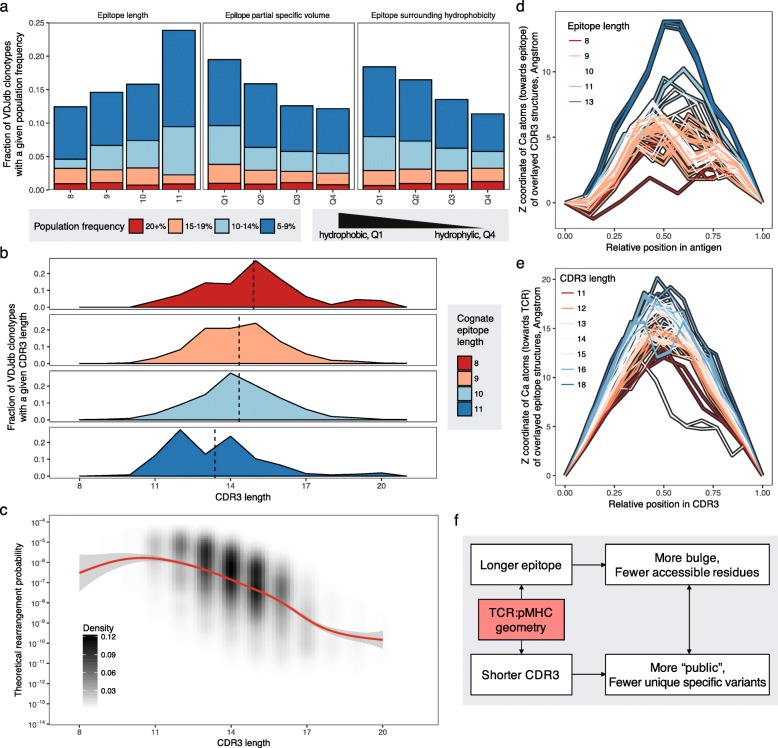
Epitope features that affect the rearrangement probabilities of specific TCRs. **a** Population frequencies of TCRs specific for epitopes of different length, net partial specific volume (sixth Kidera factor), and net surrounding hydrophobicity (tenth Kidera factor). Fractions of public clonotypes (found in 5%+ of samples) are shown with population frequencies as in Fig. 2b. The association and correlation between these features and the theoretical rearrangement probabilities is highly significant: *P*_ANOVA_ = 10^−8^, *P*_corr_ = 4 × 10^−6^ for length; *P*_ANOVA_ = 8 × 10^−9^, *P*_corr_ = 10^−6^ for partial specific volume; *P*_ANOVA_ = 4 × 10^−10^, *P*_corr_ = 4 × 10^−8^ for surrounding hydrophobicity (*P* values were corrected for multiple testing using the Benjamini–Hochberg method). Only epitope lengths of 8 to 11 amino acids were considered in the first subplot, as other lengths were represented by fewer than 30 TCRs. Partial specific volume and surrounding hydrophobicity were categorized into four quantiles (Q1 to Q4, from smallest to largest standardized value) according to their levels among VDJdb epitopes. See main text for details of feature selection. **b** CDR3 length distributions for epitope lengths of 8 to 11 amino acids. **c** Density plot of rearrangement probabilities for VDJdb TCRs with different CDR3 lengths. **d**, **e** Projection of epitope and CDR3 structures on a plane passing through the line connecting their C- and N-terminal residues and the center of mass of all Cα atoms. Longer epitope and CDR3 sequences result in more bulged structures. Data were obtained from a manually curated list of 125 PDB structures [https://github.com/antigenomics/tcr-pmhc-study]. **f** Schematic representation of the association between CDR3 and epitope lengths and the potential consequences for TCR cross-reactivity and specificity

To explore the source of large differences in the baseline frequencies of specific TCRs across epitopes, we analyzed the underlying amino acid sequence features of epitopes present in VDJdb, focusing on epitope lengths and their physicochemical properties modelled by sums of ten Kidera factors [27]. We performed correlation analysis and ANOVA. For the latter, values of each Kidera factor were categorized into four quantiles. Epitope length, net partial specific volume, and net surrounding hydrophobicity were significantly associated with specific TCR frequency (*P* < 0.01 after Benjamini–Hochberg correction for both correlation and ANOVA; Fig. 3a). Of note, the latter two Kidera factors were independent of epitope length (*P* > 0.2 for net partial specific volume, and *P* > 0.49 for net surrounding hydrophobicity, one-way ANOVA). Moreover, significant associations were observed between these Kidera factors and baseline frequencies of specific TCRs (adjusted *P* < 0.01, one-way ANOVA) when the analysis was restricted to an epitope length of 9 amino acids (the most frequent epitope length in VDJdb). Although epitope length and partial specific volume were not described previously in this context, multiple studies have suggested that hydrophobicity is an important feature related to epitope immunogenicity and TCR–peptide–MHC interactions [21, 29].

The correlation between epitope length and baseline frequencies of specific TCRs is especially interesting in the context of a recent study, which demonstrated that TCR specificity is restricted by epitope length [30]. This observation can be explained by structural constraints on the corresponding TCR–peptide–MHC interactions. Specifically, we observed that longer epitopes were recognized by TCRs with shorter CDR3 loops and vice versa (Fig. 3b) and that shorter CDR3 sequences were easier to assemble during VDJ rearrangement (Fig. 3c). Structural constraints then follow from the fact that longer CDR3 loops and epitopes are more bulged (as can be seen from the structural data analysis shown in Fig. 3d, e), such that a certain balance of CDR3 versus epitope lengths is required to allow tight docking of specific TCRs onto cognate peptide–MHC complexes. Tight docking in the context of longer epitopes may also limit the amino acid positions available for cognate TCR interactions. Following the logic shown in Fig. 3f, we can further hypothesize that longer epitopes are generally recognized by more public and less specific repertoires of TCRs.

### Exploring HLA-mediated effects on specific T cell frequencies

**Fig. 4:**
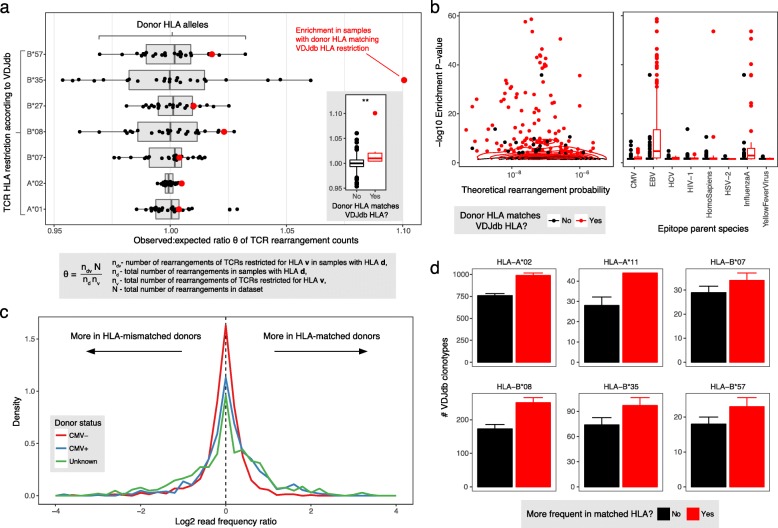
HLA-mediated selection of TCRs and epitope-specific clonal expansions. **a** Box and swarm plots show the distributions of ratios of the observed and expected numbers of rearrangements for different combinations of donor HLAs (according to genotypes from Emerson et al.) and HLAs associated with specific TCRs (according to epitope restrictions from VDJdb). Each dot represents the ratio of the total number of TCR rearrangements specific for epitopes restricted by a given HLA and the expected number of TCR rearrangements, computed with the assumption of independence between TCR restriction and donor HLA (see insert with formula). Red dots indicate matches between donor HLAs and rearranged TCRs. The inset box plot shows observed to expected ratios for matched and mismatched HLAs (**, *P* = 0.004, Mann–Whitney *U* test). Only HLA alleles present in at least 30 immune repertoire samples with at least 100 associated TCR sequences in VDJdb were selected. **b** Log10-transformed *P* values for VDJdb TCR enrichments in groups of samples with different HLAs (computed using a hypergeometric test comparing the number of times a given TCR was found in samples with and without a certain HLA). Left panel: enrichment *P* values plotted against rearrangement probabilities for sample groups that either do (red dots) or do not (black dots) have an HLA matching a given TCR (*P* > 10^−4^ shown with density plot). Right panel: the same data with epitopes grouped by source. *P* values were adjusted for multiple testing using the Benjamini–Hochberg method (TCRs with *P*_adjusted_ > 0.05 were filtered out). **c** Distribution of the log2 read frequency ratios of CMV-specific clonotypes in HLA-matched and HLA-mismatched samples from CMV-seronegative (CMV^−^, red), CMV-seropositive (CMV^+^, blue), and CMV-indeterminate donors (Unknown, green). As in previous panels, HLA matching indicates the presence of at least one HLA corresponding to the restriction element for a given TCR. All three distributions are significantly different: *P* = 6 × 10^−11^ for CMV-seropositive versus CMV-seronegative donors; *P* = 4 × 10^−4^ for CMV-seropositive versus CMV-indeterminate donors; *P* = 8 × 10^−13^ for CMV-seronegative versus CMV-indeterminate donors; Kolmogorov–Smirnov test. **d** Numbers of EBV-specific clonotypes constituting higher or lower fractions of reads in HLA-matched versus HLA-mismatched samples. Only HLA alleles associated with EBV-specific clonotypes according to VDJdb are shown (HLA-B*44 was discarded, as it was represented by just three sequences). Error bars show 95% confidence intervals (binomial distribution)

Thymic selection allows the passage of T cells that recognize peptides bound by donor HLA molecules (positive selection), yet do not interact strongly with self-peptides (negative selection) [31]. The complex interplay between positive and negative selection is therefore shaped by the ability of a TCR to bind certain HLA molecules and the pool of self-peptides presented by the donor-specific array of HLA molecules. Defining an HLA-specific TCR sequence as a TCR sequence known to recognize at least one epitope in a given HLA context according to VDJdb, we computed the extent of positive selection as the degree of association between donor HLAs and specific HLA-restricted TCRs (Fig. 4a). We detected a significant (*P* = 0.004) association between donor HLAs and the frequencies of TCRs that recognize specific epitopes in a matched HLA context, yet the effect size of this association was very small (1.02-fold increase on average). This observation suggests that HLA restriction plays a minimal role in thymic selection of the functional TCR repertoire, as described previously at the protein level [32]. As a consequence, T cells are free to recognize both HLA-matched and HLA-mismatched epitopes, which is highly pertinent in the setting of allogeneic stem cell transplantation.

To confirm this finding, we used a hypergeometric test to compare the frequencies of specific TCRs in samples with and without the corresponding HLA allele, as listed in VDJdb (Fig. 4b). We found no association between the probability of enrichment in any given HLA context and the probability of TCR rearrangement (Fig. 4b, left panel), suggesting minimal bias as a function of under-sampling certain TCRs. However, we also found that the vast majority of significantly enriched TCRs were present in samples carrying an HLA allele matching the restriction element reported in VDJdb (Fig. 4b, left panel). Of note, TCR enrichment was most prominent for epitopes derived from EBV and influenza virus (Fig. 4b, right panel).

The effect of HLA restriction on T cell selection should not be confused with HLA-restricted clonal expansions, which can be quantified by comparing sequence read frequencies (Fig. 4c, d). This phenomenon was clearly demonstrated in CMV-seropositive versus CMV-seronegative donors (Fig. 4c). As the vast majority (almost 90%) of individuals are infected with EBV by adulthood [33], one can also expect to observe HLA-restricted expansions of EBV-specific clonotypes (Fig. 4d). In line with this expectation, EBV-derived epitope-specific clonal expansions were highly discriminatory for certain HLA alleles (Additional file 1: Figure S1), explaining the accuracy of an HLA classification technique that relies on the detection of certain “predictor” TCR sequences [7].

### Umbilical cord blood is enriched for known antigen-specific TCR variants

Umbilical cord blood (UCB) contains predominantly naive but fully functional T cells that shape the TCR repertoire early in life [3, 34]. Previous studies have shown that antigen-specific TCR repertoires in UCB samples are distinct from those in peripheral blood mononuclear cell (PBMC) samples [15], featuring lower numbers of N-bases and higher numbers of public TCRs [3]. Moreover, T cells of fetal origin persist in an individual for long periods of time, with a half-life of approximately 42 years [35]. These substantial differences in repertoire structure between T cells derived from UCB and PBMC samples prompted a suggestion that these populations may also differ with respect to the recognition of certain epitopes, potentially affecting immune competence. We have therefore used our framework to quantify the epitope specificity profile of T cells in UCB versus PBMC samples.

Comparison of the fraction of unique TCR rearrangements matched with VDJdb records in samples from Britanova et al. showed that UCB samples contained ~ 1.3 times more specific TCR matches than PBMC samples (*P* = 0.0015, two-tailed *T* test, Additional file 1: Figure S2). This difference could not be attributed to the CD4/CD8 ratio bias in UCB samples, because the same effect was observed for HLA class II-restricted epitopes (*P* = 0.0008, two-tailed *T* test; Additional file 1: Figure S2). The probable explanation here is that UCB clonotypes are more likely to be observed in antigen-specific responses as a function of simpler rearrangements and prolonged persistence. Moreover, there were notable differences between the specificity profiles observed in UCB versus PBMC samples. In particular, the relative abundance of specific TCR rearrangements was significantly different for 7 of 33 epitopes (Additional file 1: Figure S3 and Table S1).

### Evidence of ancestry-associated differences in baseline frequencies of specific T cells

Ancestry is a major determinant of population-specific differences in susceptibility to immune-related diseases and various pathogens [36, 37]. In line with these observations, previous studies have documented ancestry-related differences in T cell immunity [38, 39]. However, to the best of our knowledge, there have been no previous attempts to link these findings to the composition of the T cell repertoire. We took advantage of the racially diverse cohort used in the Emerson et al. study to explore this possibility. For 9 of 33 epitopes, there was a significant variance in TCR frequencies across individuals of Caucasian, African, and Asian descent (Additional file 1: Figure S4 and Table S2). These results suggest that substantial differences may exist among populations with respect to T cell antigen specificity.

### Linking specific T cell frequencies and epitope immunogenicity

A recently published study [21] provided a large set of immunogenic and non-immunogenic epitopes, allowing us to test for an association between epitope-specific TCR frequency and immunogenicity. Epitope immunogenicity is not defined in VDJdb. However, it is still possible to score immunogenicity on a continuous scale, either by comparing the distance between each epitope and those categorized as immunogenic or non-immunogenic in the Chowell et al. dataset with respect to discriminatory features in amino acid sequence space or by training an immunogenic epitope classifier and using it to compute “immunogenicity” scores.

**Fig. 5:**
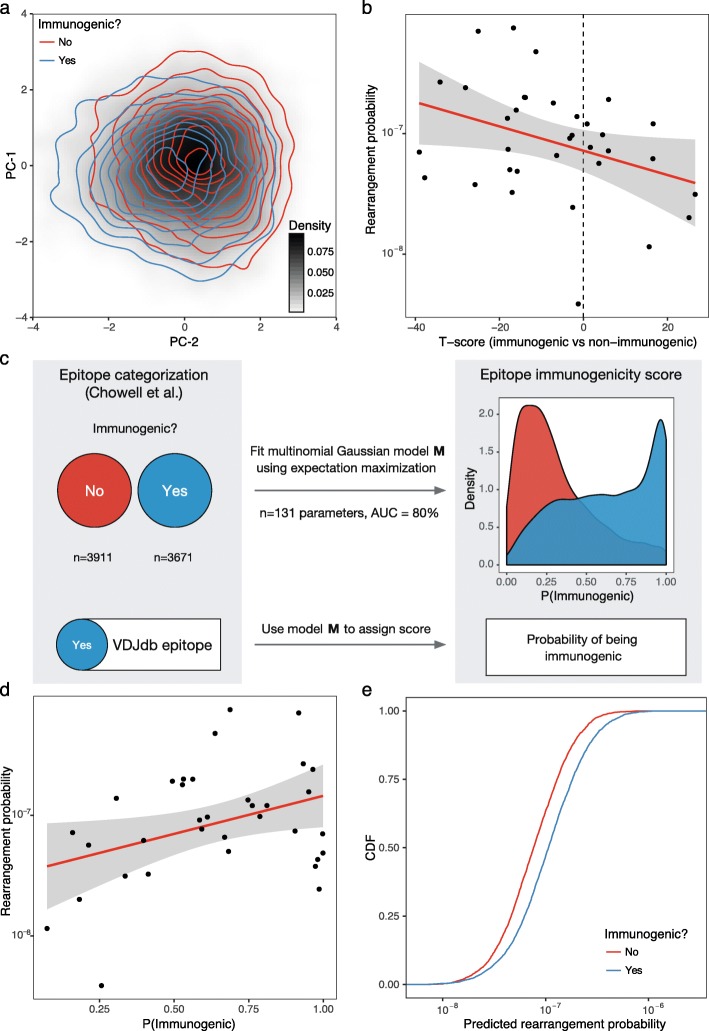
Specific T cell frequencies at baseline correlate with epitope immunogenicity profiles. **a** Principal component analysis of epitope space for immunogenic and non-immunogenic epitopes from Chowell et al. Dimensionality reduction was performed on 10-dimensional vectors of Kidera factor sums for each epitope, and the first two principal components were used to plot each epitope into a 2D plane using the Euclidean distance between Kidera factor vectors. The density map shows the overall epitope repertoire space. Red and blue contour maps show densities for immunogenic and non-immunogenic epitopes, respectively. **b** Correlation of median theoretical rearrangement probabilities of TCRs specific for certain epitopes and T-scores for the Euclidean distance of each VDJdb epitope to the immunogenic and non-immunogenic epitopes computed in Kidera factor space (*R* = 0.35, *P* = 0.039). T-scores were computed by comparing distances from a given epitope to immunogenic versus non-immunogenic epitopes. Only epitopes with more than 30 associated TCRs were selected from VDJdb. **c** A schematic representation of the algorithm used to transform categorical representation of immunogenicity (yes/no for data from Chowell et al., and yes/unknown for VDJdb epitopes) into a continuous set of probability values using an immunogenicity classifier to enable a correlation analysis between immunogenicity and TCR repertoire structure. **d** Correlation of median theoretical rearrangement probabilities of TCRs specific for certain epitopes and the probability of a given epitope being immunogenic as estimated using an expectation maximization classifier (*R* = 0.37, *P* = 0.031). **e** Cumulative distribution function plot for median rearrangement probabilities predicted for immunogenic and non-immunogenic epitopes using a simple linear model based on Kidera factor sums. The difference in predicted values for all data from Chowell et al. is highly significant (*P* < 2 × 10^−16^, Kolmogorov–Smirnov test)

Immunogenic and non-immunogenic epitopes were efficiently separated in Kidera factor feature space by transforming every epitope sequence into a vector of sums for each of the ten Kidera factors that encode the physicochemical properties of amino acids (Fig. 5a). As can be seen from Fig. 5b, c, theoretical epitope-specific T cell frequencies estimated using our models correlated positively with VDJdb epitope similarity to those defined as immunogenic by Chowell et al. and with the probabilities of VDJdb epitopes being classified as immunogenic, which in turn correlated positively with the median rearrangement probabilities determined for the corresponding epitope-specific TCRs (Fig. 5d). Conversely, when using the link between epitope features and TCR frequencies introduced previously (Fig. 3) and predicting TCR frequencies using a simple linear model (log TCR frequency fit using values of ten Kidera factor sums) for the Chowell et al. data, we found that significantly higher TCR frequencies were predicted for immunogenic epitopes (Fig. 5e). It is also important to note that higher TCR frequencies were associated with epitopes located closer to the “core” set of immunogenic epitope sequences (i.e., inside a denser region of immunogenic epitope feature space) (Additional file 1: Figure S5). Thus, a degree of variance can be expected in the T cell “view” of epitopes defined as immunogenic on the basis of physicochemical determinants.

### Concerning the effect of missing TCRα chain information on the overall analysis

**Fig. 6:**
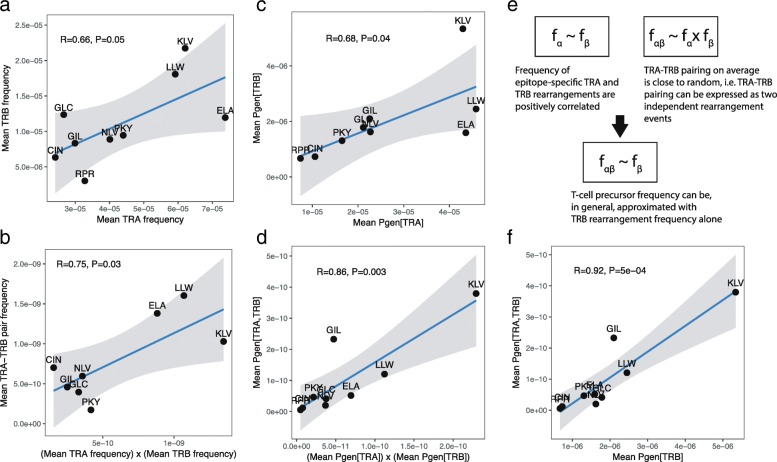
Epitope-specific TCRα-TCRβ heterodimer frequencies can be estimated using TCRβ clonotype frequencies. **a**, **b** Matching paired TCRα-TCRβ sequencing data (PairSEQ assay, Howie et al.) against VDJdb. **a** Scatter plot of TCRα and TCRβ chain rearrangement frequencies matching a given epitope. **b** Product of marginal frequencies of TCRα and TCRβ chain rearrangements (i.e., TCR heterodimer frequencies assuming independent pairing) plotted against the frequencies of paired-chain records matching the same epitopes. Mean frequencies were computed as follows: (number of matching rearrangements)/(number of records in VDJdb for a given epitope)/(total number of rearrangements in the PairSEQ dataset). **c** As in **a**, but using TCRα and TCRβ frequencies estimated using the TCR rearrangement model. **d** As in **b**, but using TCRα and TCRβ frequencies estimated using the TCR rearrangement model. **e** Conditions required to estimate baseline T cell frequencies using TCRβ rearrangement frequencies alone. **f** Scatter plot of the mean theoretical rearrangement probabilities for TCRβ chain and paired TCRα-TCRβ chain rearrangements matching a given epitope. Epitopes lacking paired TCRα-TCRβ sequences, as well as epitopes represented by less than 30 TCRα or TCRβ sequences according to VDJdb, were excluded from the analysis. This figure uses 3-letter epitope abbreviations (see Additional file 1: Table S3 for full epitope names)

One caveat of our study is that it does not account for the paired TCRα chain. This limitation stems from the fact that most of the sequence data available in the public domain were generated via bulk analyses and largely restricted to the TCRβ chain, which nonetheless allow an empirical assessment of clonotypically distributed TCRs. It is clear from previous studies that TCRα chain bias dictates immune recognition of several epitopes, such as HLA-A*02-ELA [15, 40]. We therefore expect that additional data from single-cell sequencing approaches and dedicated methods for paired-chain TCR sequencing will lead to substantial improvements in our ability to estimate baseline antigen-specific TCR frequencies [41, 42]. To assess the validity of our approach in this light, we conducted similar analyses using PairSEQ data [42]. As can be seen from Fig. 6a, b, there was a significant correlation between epitope-specific TCRα and TCRβ chain rearrangement frequencies, and paired TCRα-TCRβ chain rearrangement frequencies approximated the corresponding independent TCRα and TCRβ chain rearrangement frequencies. These results were reproduced using the TCR rearrangement model (Fig. 6c, d), suggesting that the estimates reported in this paper can be extrapolated to TCRα chain and paired TCRα-TCRβ chain data (Fig. 6e, f).

## Discussion

Numerous studies have shown that antigen presentation by MHC molecules is a critical determinant of immunogenicity [24, 25, 43]. More recently, advances in the field of immune repertoire informatics [4, 5, 44–46] have allowed us to look at this problem from another angle, taking the perspective of the host immune system represented by an array of specific TCRs. In this study, we used TCR repertoire sequencing data to investigate how antigen-specific T cells discriminate among epitopes presented by HLA class I molecules.

The baseline frequencies of antigen-specific T cells were found to vary substantially from epitope to epitope in tight linkage with the presence of public TCRs. Across individual epitopes, these frequencies varied by several orders of magnitude, in line with previous estimates based on the use of MHC multimers [12, 15, 34]. For each epitope, we observed both extremely common and extremely rare TCRs. Of note, we did not find greater numbers of public TCRs specific for epitopes derived from common pathogens, such as CMV and EBV, although we did find that amalgamated clonotype frequencies varied considerably among pathogens, grouping epitopes by source. However, this latter finding should be treated with caution, because VDJdb still lists a relatively small fraction of known epitopes for each host species.

Immune repertoire diversity can be computed in various ways [5, 47, 48]. Although used with a degree of success in several RepSeq studies, the number of unique T cell clones (either observed in a sample or estimated to be present in the entire repertoire) is in no way the same as the number of antigen specificities encoded in the overall repertoire. This limitation can be solved by moving to the concept of “functional” diversity [2, 14], which accounts for the similarity of TCR sequences and their antigen binding profiles. Moreover, while the TCRβ repertoire of an individual can feature more than ~ 10^9^ unique clones [14], the probability of a specific T cell encountering an antigen-presenting cell bearing a cognate epitope is proportional to the fraction of specific cells rather than the number of specific TCR variants. Thus, although we sampled just a minor fraction of each individual T cell pool (~ 10^6^ from up to ~ 10^12^ individual T cells) and could not accurately estimate the total diversity of the T cell repertoire, this did not limit our ability to estimate baseline frequencies and functional diversity. It is also important to note that VDJdb lists only a sample of specific clonotypes for each epitope, but again, this likely did not introduce significant bias into our median baseline frequency estimates, because there was no correlation between these frequency estimates and the number of epitope-specific TCRs (Additional file 1: Figure S6). In addition, potential confounders lurked in the origins of the RepSeq data, which were generated using bulk PBMCs. The resulting sequences therefore emanated from both naive and antigen-experienced T cells. As a consequence of clonal expansion, the latter almost invariably contribute the majority of sequence reads, but these sequences generally represent just a small fraction of the total number of unique rearrangements [3]. Accordingly, our approach most probably yielded results’ characteristics of the naïve T cell compartment, because we focused on counting unique TCRs.

Studying the incidence of T cells specific for different epitopes across the repertoires of individuals with different HLA genotypes can provide insights into the behavior of the cellular immune system during transplantation. The enrichment observed for TCRs known to engage certain HLA class I molecules in HLA-matched samples highlights the effect of positive selection in thymus. However, the effect size of this phenomenon was dwarfed by the magnitude of HLA-restricted clonal expansions observed for specific epitopes derived from CMV or EBV. We can therefore speculate that positive selection in the thymus is more focused on general features of TCR–peptide–MHC interactions rather than the specific features of individual HLA molecules. As shown previously [3, 20], the naive T cell repertoire was highly similar across individuals with respect to the relative abundance of public TCR variants, including those inherited from the fetal period [35]. We also detected T cells known to recognize certain HLA class I molecules at just slightly lower frequencies in HLA-mismatched versus HLA-matched donors. Collectively, these findings suggest a high degree of HLA cross-reactivity, in line with an overall requirement to cover the universe of potential antigens within a limited individual framework of germline-encoded antigen-presenting molecules [49, 50].

Three other features of our analysis are particularly noteworthy. First, we identified differences in the TCR specificity profiles of repertoires isolated from UCB versus PBMC samples. Substantial fractions of T cells specific for all surveyed epitopes were nonetheless present in UCB samples, highlighting the remarkable pre-immune reservoir of virus-specific T cells [51]. This result demonstrates the capability of our analytical framework to identify different T cell populations and reveals potential differences in immune coverage among T cells derived from UCB and PBMCs. Second, we found differences in the baseline frequencies of specific T cells across individuals with different ancestries, potentially indicating genetic variance in the VDJ rearrangement machinery and/or thymic selection in the context of different HLA molecules. Although further studies are required to characterize this phenomenon in more detail, such differences may have important consequences for population-level immunity and rational vaccine design. Of note, we did not find any significant gender-related differences using data from the Britanova et al. and Emerson et al. studies. Finally, we refuted a long-standing concern that analyses reliant on TCRβ sequence data alone are inherently uninformative or biased, at least for the purposes of our study. Indeed, both TCRα and TCRβ chain frequencies specific for a given epitope were concordant, allowing the use of TCRβ sequences in isolation to derive meaningful conclusions regarding the antigen-specific landscape of heterodimeric TCRs.

## Conclusions

In summary, our data indicate that the pre-immune landscape of antigen-specific T cells is a major determinant of epitope immunogenicity. As the numbers of annotated epitopes and cognate TCR sequences deposited in the VDJdb database continue to grow, we expect that our ability to characterize novel antigens in terms of immunogenicity will increase rapidly. In addition, we note that our work provides proof-of-concept for a new type of analysis that combines high-throughput T cell repertoire sequencing and in silico testing of TCR sequences across a wide range of antigen specificities to inform our basic and translational understanding of adaptive immune reactivity.

## Additional file

Additional file 1: Supplementary Figures S1-6 and Tables S1-3. (PDF 1397 kb)

## Abbreviations

CDR3: Complementarity-determining region 3 of the T cell receptor
CMV: Cytomegalovirus
EBV: Epstein–Barr virus
HCV: Hepatitis C virus
HIV: Human immunodeficiency virus
HLA: Human leukocyte antigen
MHC: Major histocompatibility complex
PBMC: Peripheral blood mononuclear cell
RepSeq: Immune repertoire sequencing technology
TCR: T cell receptor
TCRα and TCRβ: Alpha and beta chains of the T cell receptor
UCB: Umbilical cord blood
VDJ: Variable, diversity, and joining segments of the T cell receptor
