# Supplementary material for: Exploring the pre-immune landscape of antigen-specific T cells
Source: Genome Med. 2018 Aug 25;10:68. doi: 10.1186/s13073-018-0577-7 (PMC6109350; doi:10.1186/s13073-018-0577-7)
Supplement: Supplementary file 1 — Supplementary Figures S1-6 and Tables S1-3. (PDF 1397 kb) [file 13073_2018_577_MOESM1_ESM.pdf]

Additional file

Supplementary figures and tables for Pogorelyy et al. “Exploring the pre-immune landscape of antigen-specific T-cells”.

Contents:Figures S1-S6 and Tables S1-S3

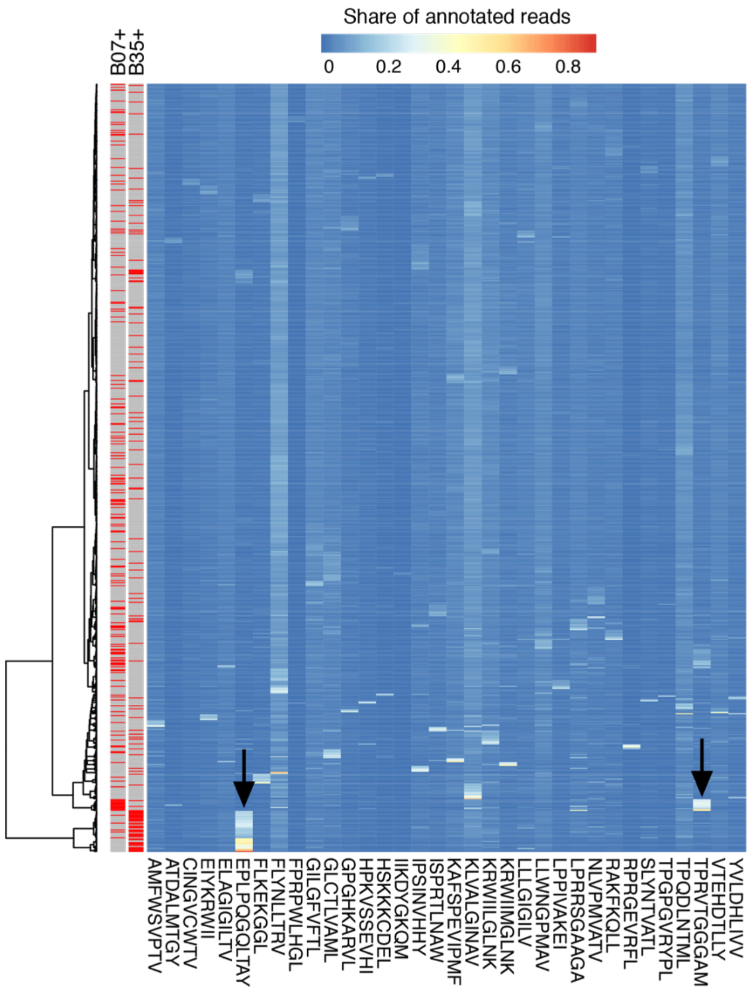

Figure S1. Heatmap of epitope-specific clonal expansions. Samples from Emerson *et al.* are clustered by the share of reads of epitope-specific TCRs (i.e. the number of TCR reads annotated with a given epitope divided by the total number of annotated reads). The graphic was generated using Ward clustering with Jensen-Shannon divergence as distance. Arrows indicate two groups of samples with prominent clonal expansions specific for HLA-B*07/EPL (EBV) and HLA-B*35/TPR (CMV). Only epitopes represented by at least 30 TCR sequences in VDJdb are shown.

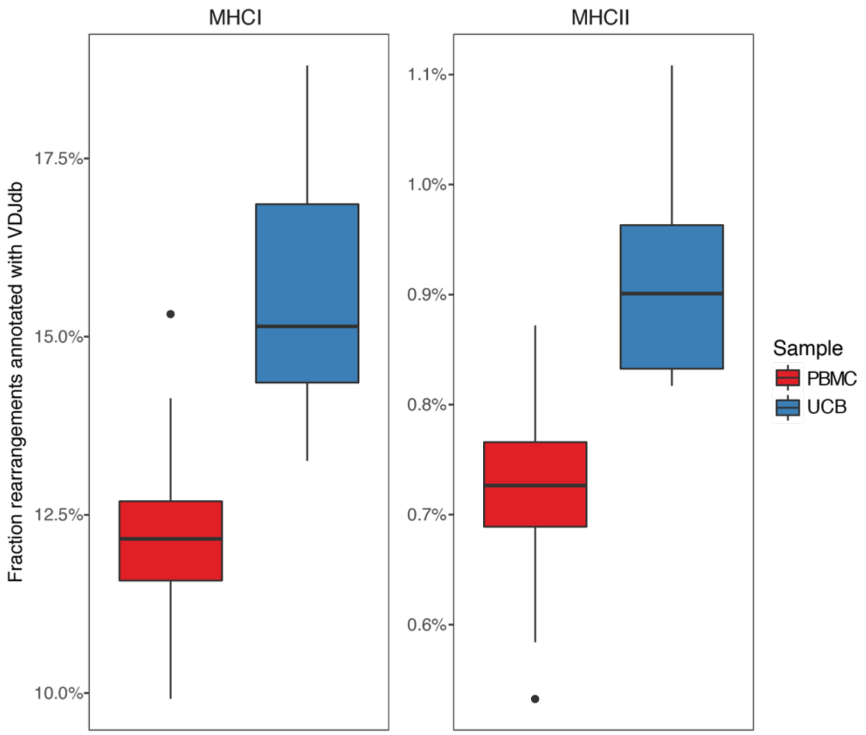

Figure S2. Fraction of annotated rearrangements in umbilical cord blood (UCB) samples versus peripheral blood mononuclear cell (PBMC) samples. The number of annotated variants is on average 1.3 times higher (P = 0.001, two-tailed T-test; values identical for HLA class I and HLA class II) in UCB samples versus PBMC samples. Samples were taken from the Britanova *et al.* study (UCB samples, n = 8; PBMC samples, n = 65; total number of TCR rearrangements, n = 29,989,055).

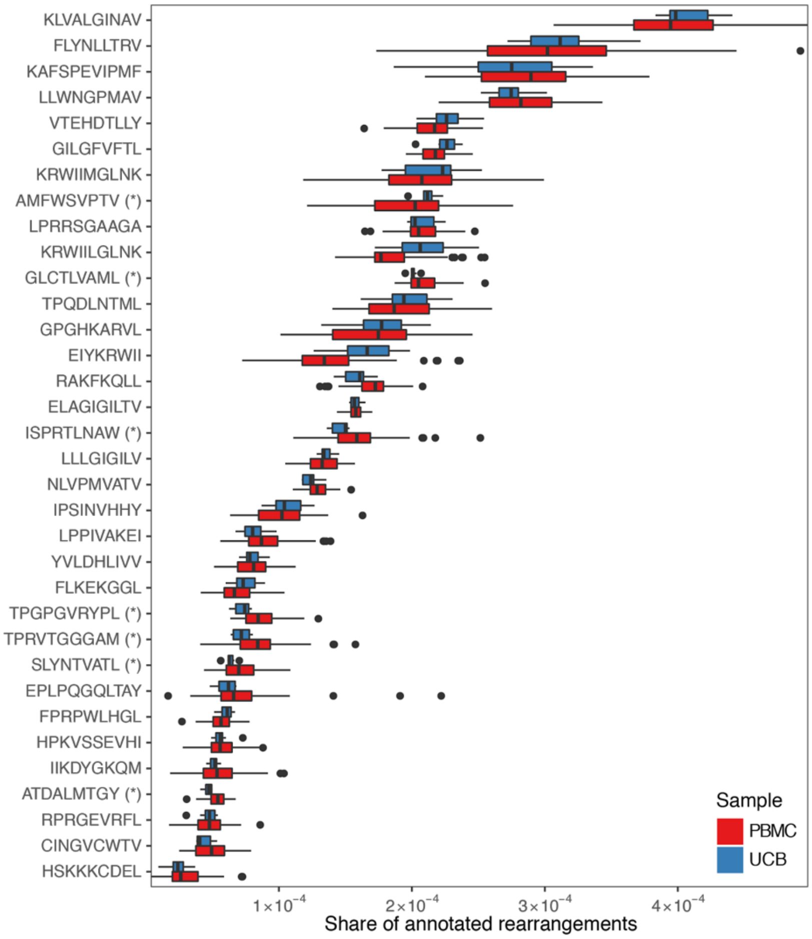

Figure S3. The relative abundance of epitope-specific TCR variants in umbilical cord blood (UCB) samples versus peripheral blood mononuclear cell (PBMC) samples. Sharing of TCR variants specific for given epitopes across all rearrangements annotated with data from VDJdb for UCB (n = 8) versus PBMC samples (n = 65). Epitopes that display a significant difference ($P < 0.05$, two-tailed T-test with Benjamini-Hochberg correction) are highlighted with asterisks. Only epitopes represented by at least 30 TCR sequences in VDJdb are shown. Samples were taken from the Britanova *et al.* study.

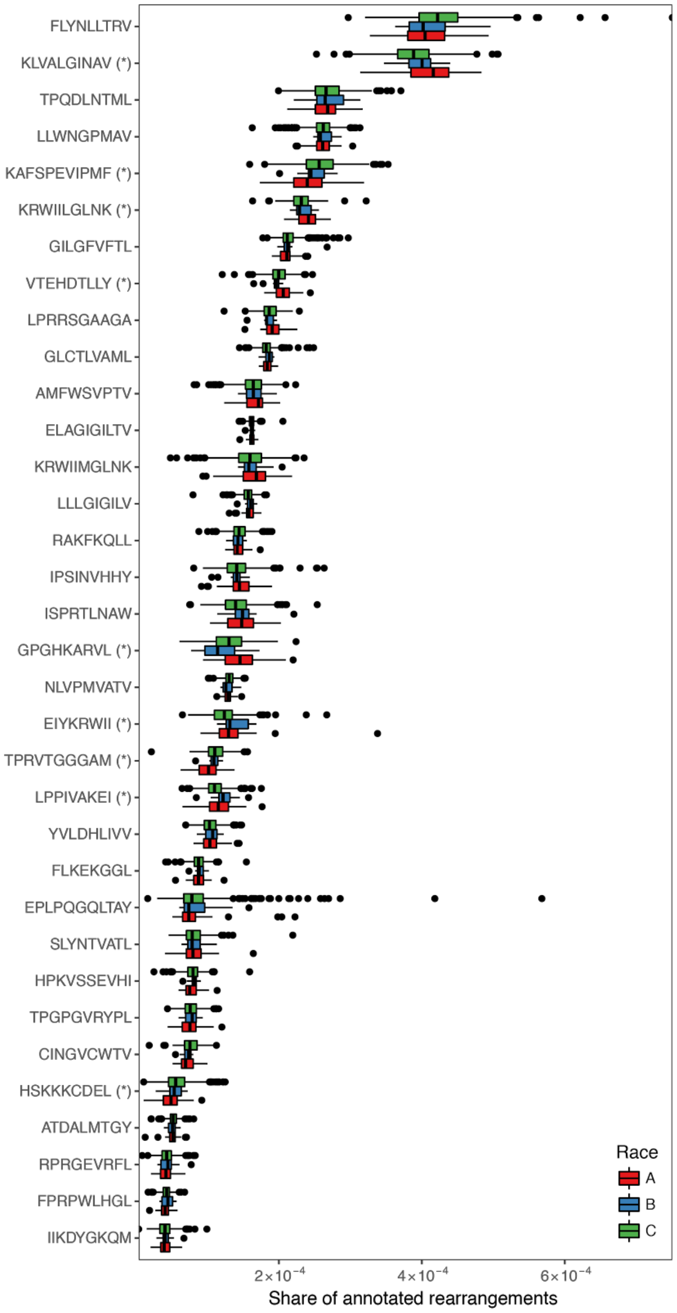

Figure S4. Sharing of epitope-specific TCR rearrangements across samples from different ancestries. Sharing of TCR variants specific for a given epitope across all rearrangements annotated with data from VDJdb for Asians/Pacific Islanders (A, red; n = 47) versus Black/African Americans (B, blue; n = 12) versus Caucasians (C, green; n = 465). Epitopes that display a significant association with ancestry ($P < 0.05$, ANOVA with Benjamini-Hochberg correction) are highlighted with asterisks. Only epitopes represented by at least 30 TCR sequences in VDJdb are shown. Samples were taken from the Emerson *et al.* study.

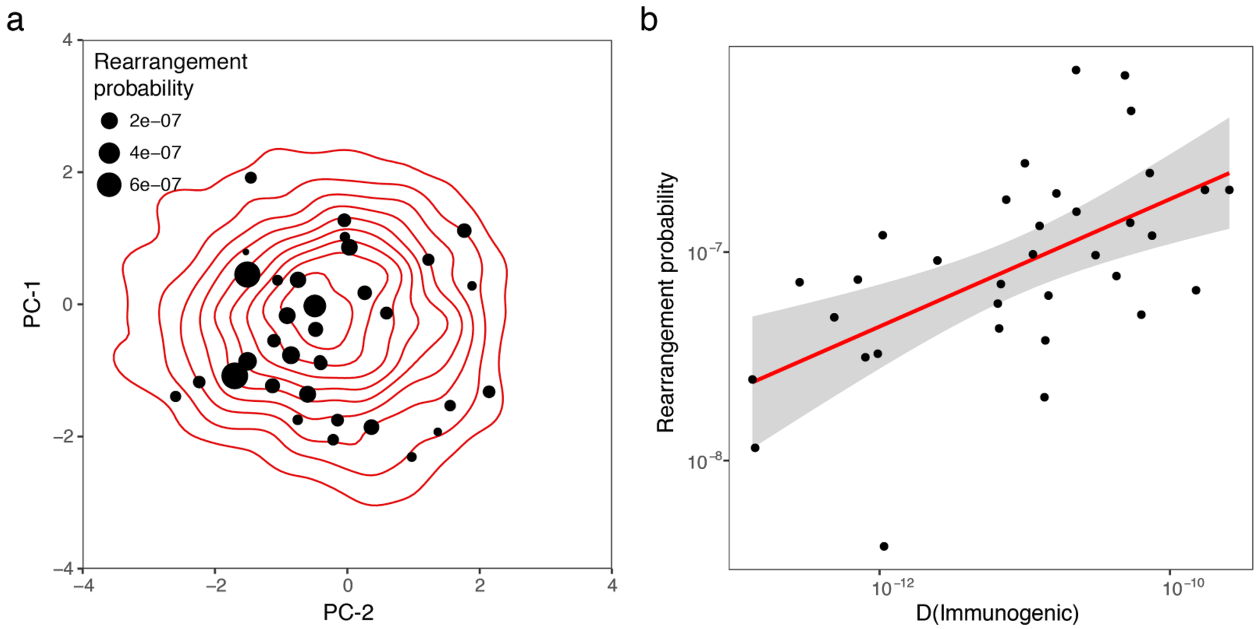

Figure S5. Epitopes associated with higher rearrangement probabilities for specific T-cells cluster closer to defined immunogenic epitopes in Kidera factor feature space. a. Principal component analysis showing the dimensionality reduction results for immunogenic epitopes (shown as a contour plot) and epitopes from VDJdb (shown as points, where point size reflects the median rearrangement probability). **b.** The density of immunogenic epitopes is higher around VDJdb epitopes with higher median rearrangement probabilities (R = 0.58, P = 4x10-4). Density was estimated using an expectation maximization classifier with Gaussian kernel (as in **Figure 5** of main text) applied to Kidera factor vectors associated with epitopes. Only epitopes represented by at least 30 TCR sequences in VDJdb are shown. Immunogenic epitopes were taken from the Chowell *et al.* study.

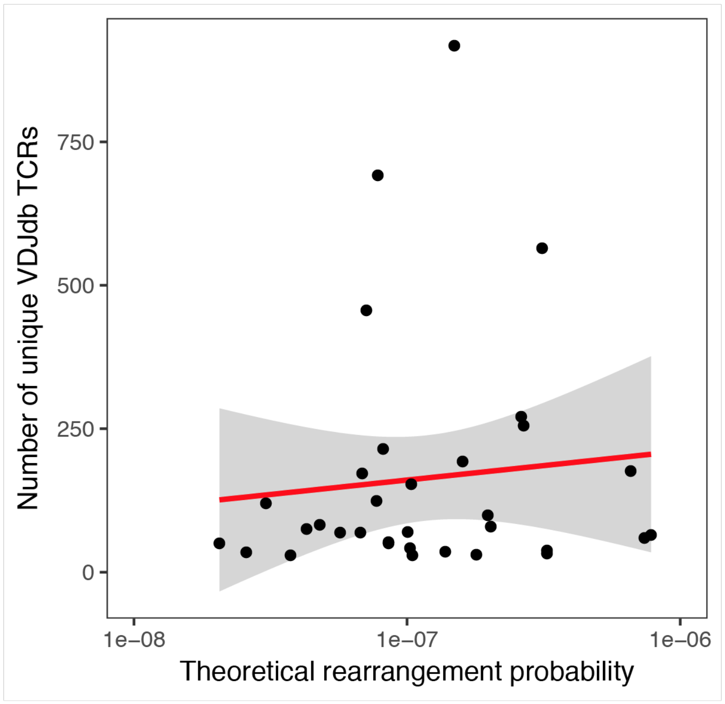

Figure S6. Differences in the numbers of annotated TCRs in VDJdb does not introduce bias in epitope precursor frequency estimates. Median rearrangement probability estimates plotted against numbers of unique variants in VDJdb for each epitope. There is no significant correlation between the number of VDJdb records and the log median rearrangement probability (R = 0.1, P = 0.59).

Table S1. Differences in relative sharing of epitope-specific T-cells in annotated TCR sequences between umbilical cord blood (UCB) and peripheral blood mononuclear cell (PBMC) samples from the Britanova *et al.* study. Only epitopes with significant differences in specific T-cell sharing between UCB and PBMC samples are shown ($P < 0.05$, two-tailed T-test with Benjamini-Hochberg correction).

**Epitope**	P-value (BH-corrected)	UCB:PBMC ratio	Species
TPGPGVRYPL	0.003	0.85	HIV-1
ATDALMTGY	0.003	0.88	HCV
TPRVTGGGAM	0.005	0.83	CMV
GLCTLVAML	0.006	0.97	EBV
SLYNTVATL	0.012	0.89	HIV-1
ISPRTLNAW	0.012	0.92	HIV-1
AMFWSVPTV	0.047	1.06	HomoSapiens

Table S2. Differences in relative sharing of epitope-specific T-cells in annotated TCR sequences between donors of different ancestries using data from the Emerson *et al.* study. Only epitopes with significant differences in specific T-cell sharing among donors of different ancestries are shown (P < 0.05, ANOVA with Benjamini-Hochberg correction).

Epitope	P-value (BH-corrected)	Ratio Caucasian:all	Ratio Black:all	Ratio Asian:all	Species
GPGHKARVL	0.003	0.99	0.89	1.10	HIV-1
KLVALGINAV	0.003	0.99	1.02	1.05	HCV
KRWIILGLNK	0.003	1.00	1.01	1.04	HIV-1
TPRVTGGGAM	0.003	1.01	0.98	0.92	CMV
KAFSPEVIPMF	0.006	1.01	0.97	0.94	HIV-1
EIYKRWII	0.009	0.99	1.11	1.07	HIV-1
HSKKKCDEL	0.009	1.02	0.95	0.83	HCV
VTEHDTLLY	0.012	1.00	0.97	1.03	CMV
LPPIVAKEI	0.016	0.99	1.11	1.04	HIV-1

Table S3. List of epitopes used in this study. The table lists epitopes and corresponding number of unique TCR beta records in VDJdb, as well as epitope parent species, corresponding MHC allele and epitope abbreviations (used in **Figure 6** of the main text). Only epitopes with at least 30 unique TCR beta records are listed. * - epitopes having both alpha and beta TCR sequencing data. ** - PKY epitope is MHCII-restricted and was only used in paired alpha-beta TCR frequency analysis shown in **Figure 6** of the main text.

Epitope	Abbreviation	Parent species	MHC allele	#unique TCRb records
ELAGIGILTV	ELA*	HomoSapiens	HLA-A*02	952
GLCTLVAML	GLC*	EBV	HLA-A*02	714
GILGFVFTL	GIL*	InfluenzaA	HLA-A*02	589
NLVPMVATV	NLV*	CMV	HLA-A*02	493
KRWIILGLNK	KRW-1	HIV-1	HLA-B*27	292
LLWNGPMAV	LLW*	YellowFeverVirus	HLA-A*02	265
LLLGIGILV	LLL	HomoSapiens	HLA-A*02	233
VTEHDTLLY	VTE	CMV	HLA-A*01	201
KAFSPEVIPMF	KAF	HIV-1	HLA-B*57	191
RAKFKQLL	RAK	EBV	HLA-B*08	179
LPRRSGAAGA	LPR	InfluenzaA	HLA-B*07	159
ATDALMTGY	ATD	HCV	HLA-A*01	131
TPRVTGGGAM	TPR	CMV	HLA-B*07	131
TPQDLNTML	TPQ	HIV-1	HLA-B*81,HLA-B*42	113
FPRPWLHGL	FPR	HIV-1	HLA-B*42	89
AMFWSVPTV	AMF	HomoSapiens	HLA-A*02	84
YVLDHLIVV	YVL	EBV	HLA-A*02	82
CINGVCWTV	CIN*	HCV	HLA-A*02	73
GPGHKARVL	GPG	HIV-1	HLA-B*07	72
KRWIIMGLNK	KRW-2	HIV-1	HLA-B*27	71
KLVALGINAV	KLV*	HCV	HLA-A*02	66
FLYNLLTRV	FLY	HomoSapiens	HLA-A*02	61
IPSINVHHY	IPS	CMV	HLA-B*35	56

ISPRTLNAW	ISP	HIV-1	HLA-B*57	56
HPKVSSEVHI	HPK	HIV-1	HLA-B*42	54
TPGPGVRYPL	TPG	HIV-1	HLA-B*42	45
LPPIVAKEI	LPP	HIV-1	HLA-B*42	44
EIYKRWII	EIY	HIV-1	HLA-B*08	38
IIKDYGKQM	IIK	HIV-1	HLA-B*42	37
SLYNTVATL	SLY	HIV-1	HLA-A*02	37
RPRGEVRFL	RPR*	HSV-2	HLA-B*07	32
EPLPQGQLTAY	EPL	EBV	HLA-B*35	31
HSKKKCDEL	HSK	HCV	HLA-B*08	31
PKYVKQNTLKLAT	PKY**	InfluenzaA	HLA-DRA*01	295
